# Prospective observational study of the efficacy of nivolumab in Japanese patients with advanced melanoma (CREATIVE study)

**DOI:** 10.1093/jjco/hyab064

**Published:** 2021-06-12

**Authors:** Naoya Yamazaki, Tatsuya Takenouchi, Yasuhiro Nakamura, Akira Takahashi, Kenjiro Namikawa, Shigehisa Kitano, Tomonobu Fujita, Kazumi Kubota, Takeharu Yamanaka, Yutaka Kawakami

**Affiliations:** Department of Dermatologic Oncology, National Cancer Center Hospital, Tokyo, Japan; Department of Dermatology, Niigata Cancer Center Hospital, Niigata, Japan; Department of Skin Oncology/Dermatology, Saitama Medical University International Medical Center, Saitama, Japan; Department of Dermatologic Oncology, National Cancer Center Hospital, Tokyo, Japan; Department of Dermatologic Oncology, National Cancer Center Hospital, Tokyo, Japan; Department of Experimental Therapeutics, National Cancer Center Hospital, Tokyo, Japan; Division of Cancer Immunotherapy Development, Advanced Medical Development Center, The Cancer Institute Hospital of Japanese Foundation for Cancer Research, Tokyo, Japan; Division of Cellular Signaling, Institute for Advanced Medical Research, Keio University School of Medicine, Tokyo, Japan; Department of Biostatistics and Epidemiology, Yokohama City University School of Medicine, Yokohama, Japan; Department of Biostatistics and Epidemiology, Yokohama City University School of Medicine, Yokohama, Japan; Division of Cellular Signaling, Institute for Advanced Medical Research, Keio University School of Medicine, Tokyo, Japan; Department of Immunology, School of Medicine, International University of Health and Welfare, Chiba, Japan

**Keywords:** nivolumab, melanoma, vitiligo, bias, product surveillance, post-marketing

## Abstract

**Background:**

Nivolumab, the anti-programmed cell death protein 1 antibody, has been approved for advanced melanoma, mainly based on evidence from Western countries. The profile of melanoma differs between Caucasian and Asian patients. This study was performed to obtain post-marketing data of nivolumab in Japanese patients with advanced melanoma.

**Methods:**

This prospective, observational study involved patients with unresectable or metastatic melanoma treated with nivolumab at dosages of 2 mg/kg every 3 weeks or 3 mg/kg every 2 weeks. The primary endpoints were objective response rate and overall survival. The secondary endpoints were progression-free survival and the objective response rate according to immune-related Response Evaluation Criteria in Solid Tumours.

**Result:**

Among 124 patients analysed, mucosal melanoma was the most common subtype, followed by acral lentiginous, nodular, superficial spreading and lentigo maligna melanoma. Response Evaluation Criteria in Solid Tumours evaluation showed an objective response rate of 17.7%. The median survival time was 15.93 months, and the 1-year overall survival rate was 66%. Outcomes were not significantly different among melanoma subtypes. Better overall survival and/or progression-free survival but not objective response rate were associated with performance status 0, lower levels of lactate dehydrogenase, C-reactive protein and neutrophil-to-lymphocyte ratio. Patients with immune-related adverse events showed a better objective response rate, 3-month landmark overall survival and progression-free survival than patients without immune-related adverse events.

**Conclusion:**

The objective response rate and median survival time in Japanese patients treated with nivolumab were lower in daily practice than the >30% and >30 months, respectively, seen in global phase III trials. The occurrence of immune-related adverse events may be a predictor for survival and response to treatment with nivolumab.

## Teaser

Efficacy of nivolumab was lower in post-marketing surveillance of Japanese patients with melanoma than in global trials. Immune-related adverse events were suggested to be efficacy predictors by 3-month landmark analysis.

## Introduction

The incidence of melanoma continues to rise, and melanoma maintains a high mortality rate worldwide. Based on global cancer statistics in 2018, the estimated mortality rate and incidence of melanoma were 60 712 deaths (crude rate, 0.8; age-standardized rate, 0.63 per 100 000) and 287 723 cases (crude rate, 3.8; age-standardized rate, 3.1 per 100 000), respectively (1). In Japan, in 2018, 654 melanoma-related deaths (age-standardized rate, 0.2) were reported (2) and ~3000 new cases of melanoma (age-standardized rate, 0.6) were estimated; these cases occurred among 25 000 new cases of all skin cancers, ~15% of which were melanoma (1,3).

Nivolumab is a fully human IgG4 monoclonal antibody to programmed cell death protein 1 (PD-1), which is an immune checkpoint receptor expressed on the T cell surface that is upregulated during activation of immune responses (4). Once PD-1 binds to its ligands, the PD-1 pathway negatively regulates the function of effector T cells. Tumour cells commonly overexpress the PD-1 ligands, PD-L1 and PD-L2, on their cell surface and can acquire immune resistance. Nivolumab binds to PD-1 and inhibits the association of PD-1 with its ligands, resulting in the release of negative immunoregulation and restoring the immune response of effector T cells to the tumour cells with acquired immune resistance (5).

Nivolumab was the first anti-PD-1 antibody approved for the treatment of malignant melanoma in Japan in July 2014; it was subsequently approved in the USA, European Union and various other countries and regions. Nivolumab is used as monotherapy and combination therapy for the first- and second-line treatment of patients with advanced and/or metastatic melanoma worldwide, mainly based on the favourable results of global phase III randomized controlled trials conducted mostly in Western countries (6–8). A phase II non-comparative study conducted in Japan also showed a high objective response rate (ORR) of 28.6 and 34.8% in 35 and 23 patients with advanced melanoma, respectively (9,10). Although the results of these Japanese clinical trials showed the efficacy of nivolumab, they were performed in small numbers of patients with limited background characteristics. Sufficient real-world data about the efficacy of nivolumab in a cohort of Japanese or Asian patients have not been generated.

The frequencies of the various clinical melanoma subtypes differ between Caucasian and Asian patients. In Japan, acral lentiginous melanoma (ALM) is the most common subtype (42% of all melanomas), followed by superficial spreading melanoma (SSM) (20%), nodular melanoma (NM) (10%), lentigo maligna melanoma (LMM) (8%) and mucosal melanoma (8%). ALM also constitutes the highest proportion of melanoma (around 50%) in Asian patients (11). In contrast, ALM is rare (1%) in the USA, where SSM is instead the most common subtype (63%). Ethnic differences are also seen in the status of *v-raf murine sarcoma viral oncogene homolog B1(BRAF)* mutation (11). The proportion of patients with melanoma harbouring *BRAF* mutation reportedly ranges from 50 to 60% in the USA, but is only about 30% in Japan. Although the results of several clinical trials have shown the efficacy of nivolumab for advanced melanoma in Asian patients, including Japanese patients, these ethnic differences appear to affect the efficacy of immunotherapy regionally (11).

This study was performed to obtain post-marketing data on the efficacy of the anti-PD1 monoclonal antibody nivolumab in Japanese patients with advanced melanoma, with the exploratory objective of identifying predictive and/or prognostic factors for efficacy.

## Patients and methods

### Patients

Eligible patients had confirmed unresectable stage III or IV melanoma (the American Joint Committee on Cancer (AJCC) 7th Edition) (12), were ≥20 years of age, had at least one measurable lesion by computed tomography (CT) and/or magnetic resonance imaging, were planning to undergo clinical treatment with nivolumab and had provided informed consent. The exclusion criteria were active infectious disease, interstitial lung disease or pulmonary fibrosis, a psychiatric illness that would limit compliance with the study requirements and pregnancy or potential pregnancy. Patients who were judged inappropriate for this study by their primary physicians were also excluded.

### Study design

This single-cohort, prospective observational study involved patients with unresectable or metastatic melanoma. Nivolumab was administered to patients in either a first- or second-line setting by intravenous infusion at a dosage of 2 mg/kg every 3 weeks or 3 mg/kg every 2 weeks, according to the dosage and administration approved in Japan. Image evaluation was performed just before, and then at 10, 19 and 28 weeks, and 1 year after the initial administration, permitting evaluation that was either early or delayed by 2 weeks, as this study was conducted in daily clinical practice. The evaluation schedule was defined in advance within the protocol.

**Figure 1.**
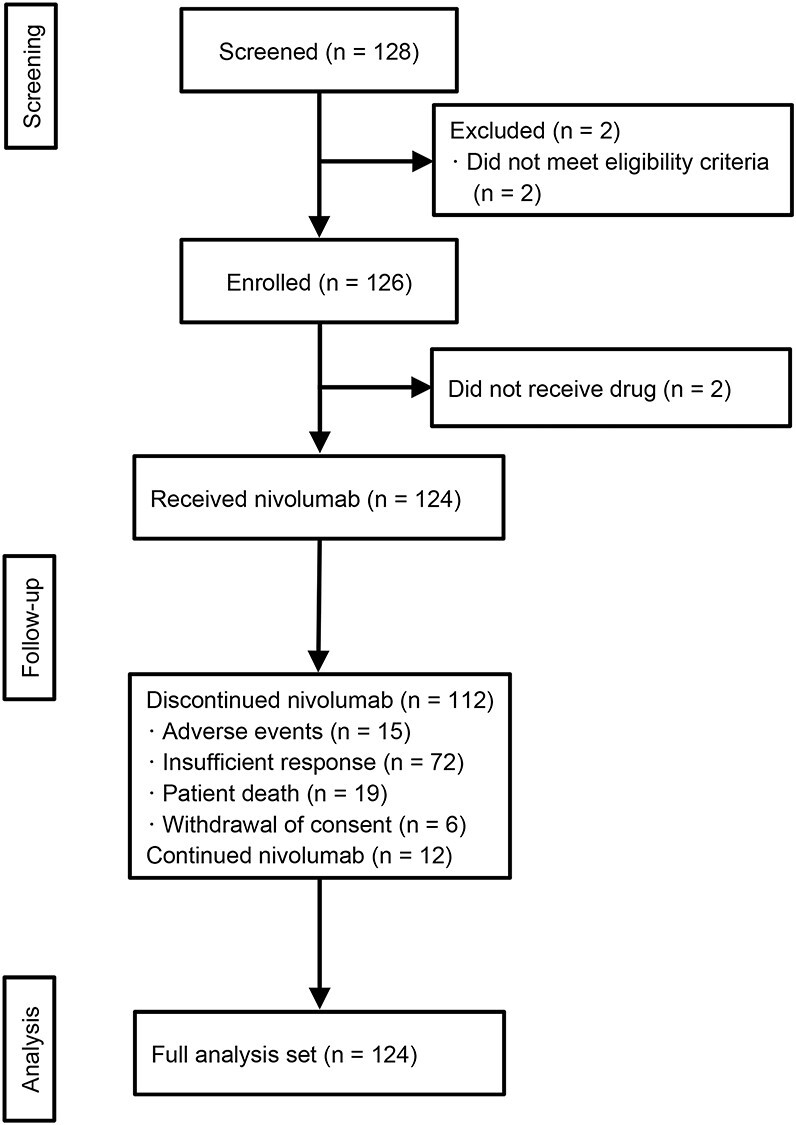
Patient flow chart.

**Table 1:** Patient demographics and clinical characteristics in the full analysis set of the CREATIVE study and the nivolumab arm in the CheckMate 037 (6), 066 (7) and 067 (18) studies

Characteristics	CREATIVE	CheckMate 037	CheckMate 066	CheckMate 067
All	*n* = 124	*n* = 272	*n* = 210	*n* = 316
Age, years				
Median [range]	65.9 [35–88]	59 [23–88]	64.0 [18–86]	58.7 [25–90]
Sex				
Male	72 (58.1)	176 (65)	121 (57.6)	202 (63.9)
Female	52 (41.9)	96 (36)	89 (42.4)	114 (36.1)
Performance status				
0	79 (63.7)	162 (60)	148 (70.5)	238 (75.3)
1	35 (28.2)	110 (40)	60 (28.6)	77 (24.4)
2	10 (8.1)		1 (0.5)	1 (0.3)
Metastasis stage				
M0, M1a, M1b	46 (37.1)	69 (25)	82 (39.0)	132 (41.8)
M1c	77 (62.1)	203 (75)	128 (61.0)	184 (58.2)
Others	1 (0.8)			
Stage				
III	12 (9.7)	11 (4)	NA	NA
IV	110 (88.7)	261 (96)	NA	NA
Others	2 (1.6)		NA	NA
LDH, IU/L				
Low^a^	92 (74.2)	132 (48.5)	120 (57.1)	196 (62.0)
High^a^	30 (24.2)	140 (51.5)	79 (37.6)	112 (35.4)
Not reported	2 (1.6)		11 (5.2)	8 (2.5)
Brain metastasis				
Absent	110 (88.7)	217 (80)	203 (96.7)	308 (97.5)
Present	14 (11.3)	55 (20)^c^	8 (3.8)^c^	8 (2.5)^c^
*BRAF* status				
Wild type	93 (75.0)	212 (78)	202 (96.2)	216 (68.4)
Mutant	20 (16.1)	60 (22)	0 (0.0)	100 (31.6)
Not investigated	11 (8.9)		8 (3.8)	
Treatment line				
First line	77 (62.1)		210 (100)	316 (100)
Second line	47 (37.9)	262 (100)		

Data are presented as *n* (%), unless otherwise indicated. NA, not available; LDH, lactate dehydrogenase.

^a^Low LDH is <400 IU/L, based on the CREATIVE study and less than equal to upper limit of normal (ULN) in global phase III studies (CheckMate 037, 066 and 067 studies), respectively.

^b^High LDH is ≥400 IU/L, based on the CREATIVE study and greater than ULN in global phase III studies.

^c^History of brain metastases without active diseases in CheckMate 037, 066 and 067 studies.

### Efficacy outcomes and assessment

The primary endpoints were ORR as assessed by investigators (investigator-assessed ORR) and overall survival (OS) (13). In this study, the investigator-assessments were conducted based on subjective judgements of the investigators. Response Evaluation Criteria in Solid Tumours (RECIST) guidelines (ver. 1.1) were used just for reference and not strictly complied with, because melanomas appear on the surface of the body such as skin or mucosa. The secondary endpoints were progression-free survival (PFS) and immune-related ORR according to the immune-related RECIST (irRECIST) guidelines (14). After primary assessments by investigators, independent radiology review committee (IRC) that consisted of two radiology experts reviewed all images and assessed the response (IRC-assessed ORR) to support investigator-assessed ORR. The immune-related ORR was assessed by investigators.

### Statistical analysis

A sample of ~200 patients was planned, assuming an ORR of 20% and median survival time (MST) of 450 days at a similar level in the previous phase II study in Japan and expecting 95% confidence intervals (95% CIs) of ±5% and ±150 days, respectively.

The efficacy analyses were performed in the intention-to-treat population. OS and PFS were analysed according to the Kaplan–Meier method. The MST and 1-year rates along with their corresponding log–log-transformed 95% CIs were derived from the Kaplan–Meier estimate. The subgroup analyses of the ORR, OS and PFS were conducted for the baseline demographics, clinical characteristics, dosing regimen, treatment line and immune-related adverse events (irAEs) (15,16). The ORRs were compared between the subgroups using a two-sided Fisher’s exact test. OS and PFS were compared between subgroups with a log-rank test. All statistical analyses were performed using SAS version 9.4 (SAS Institute, Cary, NC, USA).

## Results

### Patients

We enrolled 126 patients from 22 institutions and observed them from December 2015 to December 2018 in Japan (Fig. 1). Of these 126 patients, two were excluded because they did not receive nivolumab. The full analysis set consisted of 124 patients. The demographic and baseline clinical characteristics and the dosing regimens are summarized in Table 1. A total of 20 patients (16.1%) had *BRAF*-mutated melanoma; 14 patients (11.3%) had brain metastasis. As other information, mucosal melanoma was the most common clinical subtype (33.9%), followed by ALM, NM, SSM and LMM.

### Primary and secondary outcomes

**Figure 2.**
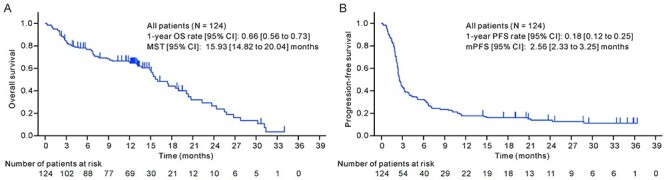
OS and PFS in the full analysis set. OS, overall survival; PFS, progression-free survival; CI, confidence interval; MST, median survival time; mPFS, median progression-free survival.

The investigator-assessed ORR and disease control rate were 17.7% (95% CI, 11.5–25.6%) and 41.1% (95% CI, 32.4–50.3%), respectively, and the IRC-assessed ORR and disease control rate were 10.5% (95% CI, 5.7–17.3%) and 23.4% (95% CI, 16.3–31.8%), respectively (Table 2). The immune-related ORR was 16.9% (95% CI, 10.8–24.7%) according to the irRECIST guidelines assessed by the investigators (Table 2). The 1-year OS rate and MST were 0.66 (95% CI, 0.56–0.73) and 15.93 months (95% CI, 14.82–20.04 months), respectively (Fig. 2A; Supplementary Table S1). The 1-year PFS rate and median PFS were 0.18 (95% CI, 0.12–0.25) and 2.56 months (95% CI, 2.33–3.25 months), respectively (Fig. 2B; Supplementary Table S2).

**Table 2:** Best response in full analysis set

	RECIST by investigators		irRECIST by investigators		RECIST by IRC	
	*n*	%	*n*	%	*n*	%
All patients	124		124		124	
CR	3	2.4	2	1.6	3	2.4
PR	19	15.3	19	15.3	10	8.1
SD	29	23.4	28	22.6	16	12.9
PD	58	46.8	60	48.4	50	40.3
NE	15	12.1	15	12.1	45	36.3
ORR	22	17.7	21	16.9	13	10.5
95% CI	11.5–25.6%		10.8–24.7%		5.7–17.3%	
DCR	51	41.1	49	39.5	29	23.4
95% CI	32.4–50.3%		30.9–48.7%		16.3–31.8%	

RECIST, Response Evaluation Criteria in Solid Tumours version 1.1; irRECIST, immune-related RECIST; IRC, independent radiology review committee; CR, complete response; PR, partial response; SD, stable disease; PD, progressive disease; NE, not evaluable; CI, confidence interval; ORR, objective response rate; DCR, disease control rate.

### Subgroup analysis of ORR

Supplementary Tables S3 and S4 show the results of the subgroup analyses of the response rate. The subgroup analyses according to the patient demographics, clinical characteristics, clinical subtype, administration regimens and treatment line revealed no significant difference in the ORR between the subgroups of patients with different subtypes (Supplementary Table S3). Patients with *BRAF* wild-type melanoma and a performance status (PS) of 0 showed better response rates than patients without these conditions, but the difference in the ORR was not statistically significant (Supplementary Table S3). Patients in first line tended to show higher response rates than those in second line (23.4 vs. 8.5%, *P* = 0.051) (Supplementary Table S4). Patients with irAEs demonstrated significantly higher ORRs than those without irAEs (*P* = 0.00003). In particular, a highly significant ORR was seen in patients who developed skin disorders (*P* = 0.0001), vitiligo (*P* = 0.0004) and thyroid dysfunctions (*P* = 0.0127) (Supplementary Table S4).

### Subgroup analysis of OS and PFS

Supplementary Tables S1 and S2 summarize the results of the subgroup analyses of OS and PFS. The subgroup analyses indicated that the clinical subtype, sex, age, *BRAF* status, brain metastasis status and treatment line did not significantly affect OS or PFS. Better OS or longer PFS was observed in patients with lower lactate dehydrogenase (LDH) level (*P* = 0.001for better OS or *P* = 0.024 for longer PFS), better PS (*P* < 0.001 for both), lower neutrophil-to-lymphocyte ratio (NLR) (*P* = 0.003 for better OS) and lower C-reactive protein (CRP) (*P* = 0.023 for longer PFS) (Supplementary Tables S1 and S2).

**Figure 3.**
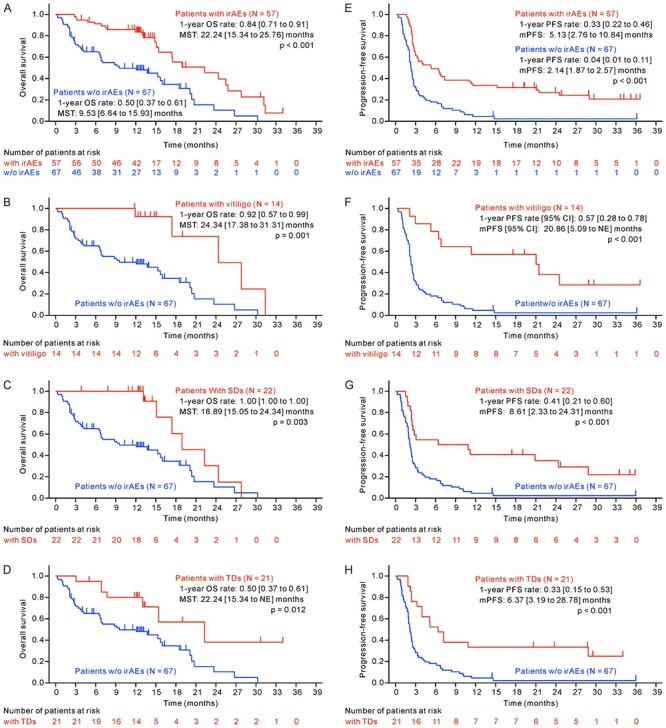
Survival analysis comparing subgroups with and without irAEs. OS and PFS were compared between patient subgroups without and with any irAEs (A and E), without and with vitiligo (B and F), without and with SDs (C and G), and without and with TD (D and H). The 1-year OS rate, MST, 1-year PFS, mPFS, and their 95% CIs [lower to upper bound] were estimated by Kaplan–Meier analysis. All *P* values were computed by a two-sided log-rank test. irAEs, immune-related adverse events; SDs, skin disorders; TD, thyroid dysfunctions; NE, not estimable.

The OS and PFS in patients who developed irAEs, including vitiligo, skin disorders and thyroid dysfunctions, were significantly better than those in patients who did not develop any irAEs (Supplementary Tables S1 and S2; Fig. 3A and E). Patients with vitiligo, skin disorders or thyroid dysfunctions showed significantly longer OS and PFS than patients with no irAEs (Fig. 3B–D and F–H). Among patients with irAEs (*n* = 57), OS and PFS were not significantly longer in patients with vitiligo (which is reportedly associated with the efficacy of nivolumab) than in patients without vitiligo (*P* = 0.244 and 0.077, respectively) (Supplementary Fig. S1).

### Landmark analyses of OS and PFS in patients with irAEs

Landmark analyses of 3-month OS and PFS were performed in patients with or without irAEs to avoid guarantee-time bias, in which analysed patients had no OS or PFS events within 3 months after the first administration of the study drug (Supplementary Fig. S2A and E). Significantly longer OS and PFS benefits were shown in patients with irAEs than in those without irAEs in these analyses (*P* = 0.024 and 0.012, respectively, by log-rank test), as shown in the ordinal OS and PFS analyses (Supplementary Fig. S2A and E). In the 3-month landmark analysis, significantly longer OS was also observed in patients with vitiligo than in patients with no irAEs (*P* = 0.016), but not in patients with skin disorders or patients with thyroid dysfunctions (*P* = 0.101 and 0.113, respectively) (Supplementary Fig. S2B–D). The landmark analysis also showed significantly longer PFS in patients with vitiligo and skin disorders than in patients with no irAEs (*P* = 0.006 and 0.002, respectively); however, the PFS benefit was only marginal in patients with thyroid dysfunctions (*P* = 0.082) (Supplementary Fig. S2F–H), which differed from the ordinal PFS analysis results.

### Classification of factors

**Figure 4.**
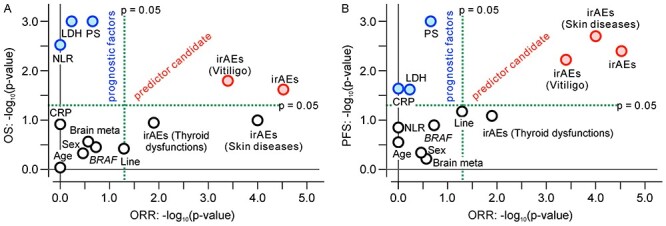
Summary of subgroup analyses to assess prognostic or predictive values for nivolumab in patients with malignant melanoma. The −log_10_ (*P* value) of the survival difference was plotted against the −log_10_ (*P* value) of the response rate difference between subgroups with and without a factor of interest in each subgroup analysis. (A) OS against ORR. (B) PFS against ORR. Notably, the *P* values in the 3-month landmark survival analysis for irAEs might have been underestimated because the sample size was smaller than in the other analyses. ORR, objective response rate; age, ≥65 versus <65 years; brain meta, brain metastasis (absent vs. present); *BRAF*, wild type versus mutation; irAEs, immune-related adverse events (patients with vs. without irAEs, OS and PFS by 3-month landmark analysis); irAEs (vitiligo, skin disorders or thyroid dysfunctions), patients with specified irAEs versus 67 patients without any irAEs, OS and PFS by 3-month landmark analysis; CRP, C-reactive protein (≥1.0 vs. <1.0 mg/dl); LDH, lactate dehydrogenase (≥400 vs. <400 IU/L); Line, treatment line (first vs. second line); NLR, neutrophil-to-lymphocyte ratio (median or greater vs. less than median); PS, performance status (0 vs. ≥1); sex, male vs. female.

The *P* values of the subgroup analyses of OS/PFS and the ORR are summarized in Fig. 4, where the −log_10_ (*P* value) of OS/PFS was plotted against the −log_10_ (*P* value) of the ORR. The *P* values in the subgroup analyses of the LDH level, PS, CRP level and NLR were plotted in the second quadrant of the charts sectioned by vertical and horizontal lines of −log_10_ (0.05) of the ORR and OS/PFS, respectively (Fig. 4A and B); namely, we observed significant differences in the survival analysis (*P* < 0.05) but not in the response rate between subgroups divided by them. The *P* values of irAEs including vitiligo and skin disorders were plotted in the first quadrant. These showed a significant difference in both survival by the 3-month landmark analysis and the response rate between the subgroups divided by them.

## Discussion

The primary endpoints of the investigator-assessed standard ORR, MST and median PFS were 17.7%, 15.93 months and 2.56 months, respectively, in the present study (Table 2; Supplementary Tables S1 and S2). These values are lower than those in the nivolumab monotherapy arm of previous global phase III studies (ORR of 27.2% assessed by IRC; ORRs of 42.9 and 43.7% assessed by investigators; and MST of 15.7, 37.5 and 36.9 months in the CheckMate 037, 066 and 067 studies, respectively) (6–8,17,18). Complete response was 2.4% for both investigator- and IRC-assessments. Also, no significant difference was observed between investigator- and IRC-assessed progressive disease (PD) (46.8 vs. 40.3%). There were, however, significant differences in partial response (PR) and stable disease (SD) between the investigator- and IRC-assessments. The investigator-assessed PR and SD were 15.3 and 23.4%, respectively, compared with the IRC-assessment (PR 8.1%; SD 12.9%). Investigator- and IRC-assessed not evaluable (NE) were 12.1 and 36.3%, respectively. The main reason for the discrepancy in ORR and DCR between the investigator- and IRC-assessments may be that many patients who have been diagnosed by the investigator to have achieved disease control (PR or SD) were diagnosed by IRC as NE, under the present circumstances where the IRC assessment was performed with confirmation according to the RECIST guidelines 1.1, whereas the investigator-assessment did not necessarily require strict confirmation. Investigators usually conduct a visual inspection of a lesion using a dermatoscope, because melanomas tend to grow as target lesions on the surface of the skin or mucosal, and it makes difficult to diagnose by CT scans. Also, if improvement in bone scan results (regeneration of damaged bones) is confirmed, investigators assess bone metastasis patients to have achieved PR or SD, without performing CT scans. These cases include lymph node metastasis with a tumour size of 10–15 mm. The immune-related ORR was 16.9% according to the irRECIST guidelines, which was not significantly different from the standard ORR (Table 2). This difference appears because of the short assessment frequency, i.e. imaging evaluation was performed in 3-month intervals in this study. Contrary to our expectations, the frequency of immune-related PD (48.4%) assessed by irRECIST was slightly higher than that of standard PD (46.8%) (Table 2). Pseudo-progression events seemed to be few in this study; these are reportedly rare events observed in 5–10% of patients receiving immune checkpoint inhibitors (19).

The frequencies of the various clinical melanoma subtypes and the proportion of patients with melanoma harbouring *BRAF* mutation differ between Caucasian and Asian patients. Subgroup analyses were performed to search for a cause of the different efficacies between patients in Japan and Western countries. ALM is the dominant histopathological subtype in Asian patients, including Japanese patients; in contrast, ALM is rare in the USA, where SSM is instead the most common (11). In the present study, however, no significant differences in the ORR, PFS or OS were detected among patients with different subtypes. The proportion of patients with *BRAF*-mutated melanoma is reportedly higher in the USA than in Japan. *BRAF*-mutated melanoma is aggressive and resistant to chemotherapy (20,21). In the present study, higher efficacy was seen in patients with *BRAF* wild-type melanoma than in those with *BRAF*-mutated melanoma. The OS was 9.53 and 15.64 months, and the ORR was 5.0 and 18.3% in patients with and without *BRAF*-mutated melanoma, respectively (Supplementary Tables S1 and S3), although the difference was not statistically significant. The CheckMate 066 study excluded patients with *BRAF*-mutated melanoma (Table 1), which might have contributed to the good response rate and survival time (7). However, a higher proportion of patients with *BRAF*-mutated melanoma were enrolled in the CheckMate 037 and 067 studies than in the present study (Table 1). We did not find expected association of histopathological subtype and BRAF mutation status with the difference between the efficacy of nivolumab in Japan and Western countries.

The lower efficacy of nivolumab in the present study does not appear referable simply to regional or racial differences because the outcomes of this study also seem to be lower than those of two previous phase II studies conducted in Japan. In one study (Japic-CTI #142533), the IRC-assessed ORR was 34.8% (8/23), the investigator-assessed ORR was 43.5% (10/23) and the MST was not reached at the median follow-up of 18.8 months (range, 2.0–21.5 months) (9). In the other study (Japic-CTI #111681), the IRC-assessed ORR and MST were 28.6% (10/35) and 18.0 months, respectively (10). Although the sample sizes were small, these results are similar to the global phase III studies mentioned above.

High baseline serum LDH and CRP levels, a high NLR and a poor PS have been reported as prognostic factors for poor survival in patients with metastatic melanoma (22–27). In our study, an LDH level of ≥400 IU/L and a PS of ≥1 were associated with shorter PFS and/or OS, but they were not associated with the ORR. In consideration of a report that LDH >1.5 × the upper limit of normal was found to be associated with worse OS in patients with malignant melanoma, 400 IU/L was selected as the cut-point for the serum LDH level (27). A CRP level of ≥1.0 mg/dl was marginally associated with shorter OS, but not with the ORR. An NLR higher than or equal to the median (2.79) was associated with shorter OS, but not with either PFS or the ORR. The LDH level, CRP level, NLR and PS did not seem to be predictive factors for nivolumab efficacy.

Because the present study was a post-marketing surveillance study including patients with a PS of ≥1, pre-treatment and brain metastasis, the ORR was lower than that in previous clinical trials, as expected. However, the OS did not seem to be inferior to the patients in past studies. Namely, the ORR was lower in this study than in the nivolumab monotherapy arm of CheckMate 037; however, the median PFS and MST were comparable (6). In addition, they were 2.56 and 15.05 months in the pre-treated patients of the present study using the same criteria as in CheckMate 037. However, large differences in the efficacy results of nivolumab monotherapy (both ORR and OS) are still present between this observational study and the CheckMate 066 and 067 studies (7,8). In a comparison of various factors between the present study and CheckMate 066/067 (Table 1), the following factors of the present study may have influenced the poorer outcomes: the higher proportions of patients with a PS of ≥1, pre-treatment and active brain metastases (Table 1). Higher efficacy of nivolumab in terms of the ORR was also seen in phase II studies in Japan (Japic-CTI #111681 and #142533) (9,10), in which a higher or equal proportion of patients had the above-mentioned poor prognostic factors with the exception of brain metastasis. Notably, however, the tumour response was assessed by an IRC, and the 1-year OS rate was 54.3 and 69.6% in those studies, whereas the OS rate was 66.0% in the present study. The actual reasons for the poorer outcomes in this study, especially with respect to the ORR, are unclear other than this study having been performed in real-world clinical practice and including patients with a poor prognosis.

A unique, immune-driven toxicity profile (i.e. the development of irAEs) is present in patients treated with immune checkpoint inhibitors. These irAEs may involve the dermatologic, gastrointestinal and endocrine systems (28,29). In this study, various irAEs were reported in 57 out of 124 patients, with skin disorders the most common (*n* = 22), followed by thyroid dysfunction (*n* = 21) and vitiligo (*n* = 14) (Supplementary Table S4).

Dermatologic irAEs are commonly observed in patients with various cancers receiving immune checkpoint inhibitors, in whom rash, pruritus and vitiligo were the most frequent dermatologic irAEs (30). The relative risk for developing dermatologic irAEs was 2.3 in patients with various cancers treated with nivolumab, and vitiligo is characteristically reported in trials investigating the use of these inhibitors in malignant melanoma (30). In addition, dermatologic irAEs are reportedly associated with a higher response rate and improved survival in patients with melanoma treated with PD-1 inhibitors, including nivolumab (28,29). Generally, dermatologic irAEs associated with nivolumab treatment are primarily low grade and manageable with established safety guidelines (7,8); therefore, the development of dermatologic irAEs seems to have a clinical benefit (29,31). In fact, the subgroup analyses in our study showed a significantly higher ORR in patients with than without dermatologic irAEs of vitiligo and skin disorders as well as longer PFS and OS in patients with these dermatologic irAEs than in patients with no irAEs, similar to previous reports (Supplementary Tables S2 and S4; Supplementary Fig. S1) (29,31).

irAEs affecting the endocrine system are frequently reported during treatment with immune checkpoint inhibitors, as are dermatologic irAEs (29,32–34). Thyroid dysfunction that includes hypothyroidism, hypophysitis, adrenal insufficiency/crises and type 1 diabetes mellitus can all occur, among which hypothyroidism is the most frequently reported endocrine irAE (32). Development of immune-related thyroid dysfunction was reportedly associated with better survival in patients with various cancers including non-small cell lung cancer, malignant melanoma and others, when treated with immune checkpoint inhibitors (33,34). However, the prognostic or predictive value of thyroid irAEs was inconclusive in malignant melanoma due to the small sample sizes in these studies (33,34). Subgroup analysis in the present study showed a significantly higher ORR in patients who developed thyroid dysfunction than in those who did not (*P* = 0.0003, *P* = 0.012 and *P* < 0.001, respectively) and significantly longer OS/PFS in patients with thyroid dysfunction than in those with no irAEs (*P* = 0.0003, *P* = 0.012 and *P* < 0.001, respectively).

The prognostic or predictive value of irAEs for survival benefit is supported by many reports (29–31,33,34), but consensus has not necessarily been reached because of the problem of survival analysis. Patients who experienced early PFS or OS events could not be counted in the irAE subgroup; they were thus classified into the subgroup of patients without irAEs, although they would have developed irAEs if they could have continued the study (guarantee-time bias or time delay bias). This provides an advantage for the subgroup of patients with irAEs in terms of the survival analysis. To avoid this bias, a 12-week landmark survival analysis has been recommended (31,35) and suggests that dermatologic irAEs alone are associated with improved survival in patients with malignant melanoma who received nivolumab (31). The present study showed a significant OS and PFS benefit in patients with vitiligo or any irAEs and a trend toward an OS and PFS benefit in patients with skin disorders and thyroid dysfunctions at the 3-month landmark analysis (Supplementary Fig. S2). Vitiligo seems to be the most reliable irAE associated with a survival benefit, based on our results and previous reports (29,31), but other irAEs also appear to be involved in the association because no significant difference was seen in OS and PFS between patients with and without vitiligo among those who developed any irAE (Supplementary Fig. S1). A further prospective study involving more patients is required to clarify the prognostic/predictive value of irAEs in nivolumab treatment.

We conducted various subgroup analyses to identify factors influencing the efficacy of nivolumab, particularly predictive factors specifically associated with the efficacy of immune checkpoint inhibitors. These analyses, however, could not separate predictive factors from prognostic factors because this was a single-arm study. The results of these subgroup analyses are summarized in Fig. 4A and B, where the −log_10_ (*P* value) of OS/PFS was plotted against the −log_10_ (*P* value) of the ORR in the subgroup analyses by each factor. The LDH, PS, CRP and NLR are typical prognostic factors that are not specific for the efficacy of nivolumab treatment (25,26), and their *P* values in the subgroup analyses were plotted in the second quadrant of the charts sectioned based on *P* = 0.05 in Fig. 4A and B; this means that they affect survival but do not affect the response rate significantly. The factors plotted in the first quadrant can provide a significant difference in both the survival and response rate between the subgroups divided by them. The predictive factors of nivolumab treatment should be plotted here because the survival effects are generally associated with tumour shrinkage in immune checkpoint inhibitor therapy. Only irAEs were plotted in the first quadrant and are thought to be candidate predictors. In patients undergoing immune checkpoint inhibitor treatment, however, predictive factors are expected to distinguish between true- and pseudo-progression events and to facilitate early selection of patients who would benefit from the treatment. irAEs may not be fully suitable for this purpose because it takes a couple of months for the development of irAEs.

The present study has several limitations. First, the lack of a control cohort makes it difficult to interpret the therapeutic potential of nivolumab in a real-world clinical setting and analyse predictive or prognostic factors. Second, the subgroup analyses involved ~30 comparisons, including various irAEs. Therefore, interpretation of the results of the subgroup analyses may require consideration of this multiplicity and a multivariate analysis to identify truly predictive or prognostic factors. Third, due to poor accrual, the sample size was 126, which was smaller than the 200 patients in the original plan. This expands the CIs of statistics and decreases the power of subgroup analyses, but does not affect the interpretation of key findings, namely that nivolumab was less efficacious in this study than it was in global phase III studies, and that we identified significant candidate predictive/prognostic factors in subgroup analyses, including irAEs. Finally, there were discrepancies in response outcomes between IRC- and investigators-assessments since many patients were assessed as NE by IRC due to insufficient CT images provided from investigators for response assessments. To reduce the discrepancies, education of the participating investigators on the response criteria is warranted in the future study in which response rate is used as a primary endpoint.

In conclusion, the results of this study of the real-world use of nivolumab showed that the ORR and survival rates in Japanese patients were lower than those reported in recent global phase III randomized trials. Although the reasons for these differences remain unclear, the efficacy of nivolumab monotherapy does not appear to be particularly high in real-world clinical practice. Treatment algorithms including patient selection and combination therapies should be improved. Patients who developed irAEs showed a tendency to achieve better outcomes in the real-world use of nivolumab, consistent with the results in previous clinical studies.

## Supplementary Material

Real-world_outcomes_of_nivolumab_in_melanoma_Suppl_Tables_and_Figs_hyab064
