# Supplementary material for: Prospective observational study of the efficacy of nivolumab in Japanese patients with advanced melanoma (CREATIVE study)
Source: Jpn J Clin Oncol. 2021 Jun 12;51(8):1232–41. doi: 10.1093/jjco/hyab064 (PMC8326387; doi:10.1093/jjco/hyab064)
Supplement: Real-world_outcomes_of_nivolumab_in_melanoma_Suppl_Tables_and_Figs_hyab064 [file real-world_outcomes_of_nivolumab_in_melanoma_suppl_tables_and_figs_hyab064.doc]

**SUPPLEMENTARY DATA**

**Prospective observational study of the efficacy of nivolumab in Japanese patients with advanced melanoma (CREATIVE study)**

Naoya Yamazaki, Tatsuya Takenouchi, Yasuhiro Nakamura, Akira Takahashi,

Kenjiro Namikawa, Shigehisa Kitano, Tomonobu Fujita, Kazumi Kubota,

Takeharu Yamanaka, Yutaka Kawakami

**Supplementary Table 1.** One-year OS rate and MST in subgroup analyses in the full analysis set

|  | n | 1-year OS rate | | MST | | p-value |
| --- | --- | --- | --- | --- | --- | --- |
|  |  | Rate | 95% CI | Months | 95% CI |  |
| All patients in FAS | 124 | 0.66 | 0.56–0.73 | 15.93 | 14.82–20.04 |  |
| Sex |  |  |  |  |  |  |
| Male | 72 | 0.60 | 0.48–0.71 | 15.34 | 8.77–22.24 | 0.469 |
| Female | 52 | 0.73 | 0.58–0.84 | 17.45 | 13.70–20.67 |  |
| Age, years |  |  |  |  |  |  |
| ≥65 | 79 | 0.64 | 0.52–0.74 | 15.64 | 14.82–22.24 | 0.911 |
| <65 | 45 | 0.69 | 0.53–0.80 | 15.93 | 13.01–20.67 |  |
| PS |  |  |  |  |  |  |
| 0 | 79 | 0.82 | 0.71–0.89 | 18.89 | 15.34–24.34 | <0.001 |
| ≥1 | 45 | 0.36 | 0.21–0.50 | 6.64 | 2.73–15.28 |  |
| BRAF |  |  |  |  |  |  |
| WT | 93 | 0.67 | 0.56–0.75 | 15.64 | 13.70–18.89 | 0.352 |
| Mutation | 20 | 0.47 | 0.24–0.68 | 9.53 | 2.66–NE |  |
| Brain metastasis |  |  |  |  |  |  |
| Absent | 110 | 0.69 | 0.59–0.77 | 15.93 | 14.82–20.04 | 0.272 |
| Present | 14 | 0.43 | 0.18–0.66 | 8.30 | 2.10–NE |  |
| LDH, IU/L |  |  |  |  |  |  |
| <400 | 92 | 0.72 | 0.62–0.81 | 17.38 | 15.05–20.67 | 0.001 |
| ≥400 | 30 | 0.43 | 0.25–0.59 | 6.97 | 2.10–18.50 |  |
| CRP, mg/dL |  |  |  |  |  |  |
| <1.0 | 79 | 0.71 | 0.60–0.80 | 17.38 | 14.82–23.62 | 0.121 |
| ≥1.0 | 39 | 0.51 | 0.34–0.66 | 13.11 | 3.68–18.89 |  |
| Treatment line |  |  |  |  |  |  |
| First-line | 77 | 0.69 | 0.57–0.78 | 17.38 | 14.82–20.04 | 0.378 |
| Second-line | 47 | 0.61 | 0.45–0.73 | 15.05 | 7.46–23.62 |  |
| NLR |  |  |  |  |  |  |
| <median (2.79) | 61 | 0.83 | 0.70–0.90 | 18.89 | 15.34–23.62 | 0.003 |
| ≥median (2.79) | 62 | 0.48 | 0.34–0.60 | 7.66 | 5.82–18.59 |  |
| irAEs |  |  |  |  |  |  |
| Absent | 67 | 0.50 | 0.37–0.61 | 9.53 | 6.64–15.93 | < 0.001 |
| Present | 57 | 0.84 | 0.71–0.91 | 22.24 | 15.34–25.76 |  |
| Histopathological subtypes | |  |  |  |  |  |
| All classified patients | 112 | 0.64 | 0.55–0.73 | 15.64 | 13.11–18.89 |  |
| Mucosal | 42 | 0.67 | 0.50–0.79 | 15.64 | 11.89–18.50 | - |
| ALM | 25 | 0.76 | 0.54–0.88 | 17.45 | 13.11–25.76 | - |
| NM | 19 | 0.56 | 0.31–0.75 | 13.70 | 5.82–NEs | - |
| SSM | 16 | 0.48 | 0.22–0.70 | 9.53 | 2.99–20.34 | - |
| LMM | 3 | 1.00 | 1.00–1.00 | NEs | NEs–NEs | - |
| OS, overall survival; MST, median survival time; NEs, not estimable; CI, confidence interval; PS, performance status; WT, wild type; LDH, lactate dehydrogenase; CRP, C-reactive protein; irAEs, immune-related adverse events; ALM, acral lentiginous melanoma; LMM, lentigo maligna melanoma; Mucosal, mucosal melanoma; NLR, neutrophil-to-lymphocyte ratio; NM, nodular melanoma; SSM, superficial spreading melanoma | | | | | | |

**Supplementary Table 2.** One-year PFS rate and median PFS in subgroup analyses

|  | n | 1-year PFS rate | | Median PFS | | Log-rank p-value |
| --- | --- | --- | --- | --- | --- | --- |
|  |  | Rate | 95% CI | Months | 95% CI |  |
| All patients | 124 | 0.18 | 0.12–0.25 | 2.56 | 2.33–3.25 |  |
| Sex |  |  |  |  |  |  |
| Male | 72 | 0.21 | 0.12–0.31 | 2.64 | 2.30–3.38 | 0.455 |
| Female | 52 | 0.13 | 0.06–0.24 | 2.51 | 2.20–3.94 |  |
| Age, years |  |  |  |  |  |  |
| ≥65 | 79 | 0.22 | 0.13–0.31 | 2.79 | 2.30–4.40 | 0.280 |
| <65 | 45 | 0.11 | 0.04–0.22 | 2.46 | 2.23–2.86 |  |
| PS |  |  |  |  |  |  |
| 0 | 79 | 0.23 | 0.14–0.32 | 3.25 | 2.56–5.13 | <0.001 |
| ≥1 | 45 | 0.09 | 0.03–0.19 | 1.87 | 1.45–2.33 |  |
| BRAF |  |  |  |  |  |  |
| WT | 93 | 0.17 | 0.10–0.26 | 2.53 | 2.33–3.78 | 0.127 |
| Mutation | 20 | 0.10 | 0.02–0.27 | 2.35 | 1.38–2.79 |  |
| Brain metastasis |  |  |  |  |  |  |
| Absent | 110 | 0.17 | 0.11–0.25 | 2.64 | 2.33–3.25 | 0.606 |
| Present | 14 | 0.21 | 0.05–0.45 | 2.27 | 1.25–10.02 |  |
| LDH, IU/L |  |  |  |  |  |  |
| <400 | 92 | 0.20 | 0.12–0.28 | 2.73 | 2.37–3.91 | 0.024 |
| ≥400 | 30 | 0.10 | 0.03–0.24 | 1.81 | 1.18–3.25 |  |
| CRP, mg/dL |  |  |  |  |  |  |
| <1.0 | 79 | 0.22 | 0.13–0.31 | 3.25 | 2.37–5.06 | 0.023 |
| ≥1.0 | 39 | 0.10 | 0.03–0.22 | 2.3 | 1.81–2.53 |  |
| Treatment line |  |  |  |  |  |  |
| First-line | 77 | 0.22 | 0.14–0.32 | 2.86 | 2.27–5.09 | 0.067 |
| Second-line | 47 | 0.11 | 0.04–0.21 | 2.56 | 2.23–2.79 |  |
| irAEs |  |  |  |  |  |  |
| Absent | 67 | 0.04 | 0.01–0.11 | 2.14 | 1.87–2.50 | <0.001 |
| Present | 57 | 0.33 | 0.22–0.46 | 5.13 | 2.76–10.84 |  |
| NLR |  |  |  |  |  |  |
| <median^a^ | 61 | 0.21 | 0.12–0.32 | 2.76 | 2.37–5.09 | 0.141 |
| ≥median^a^ | 62 | 0.13 | 0.06–0.22 | 2.33 | 2.07–3.25 |  |
| Histopathological classification | | |  |  |  |  |
| All classes | 112 | 0.18 | 0.11–0.25 | 2.51 | 2.33–3.19 |  |
| Mucosal | 42 | 0.12 | 0.04–0.24 | 2.38 | 2.07–3.02 | - |
| ALM | 25 | 0.16 | 0.05–0.33 | 3.25 | 2.33–9.53 | - |
| NM | 19 | 0.21 | 0.07–0.41 | 2.56 | 1.87–3.38 | - |
| SSM | 16 | 0.25 | 0.08–0.47 | 2.45 | 1.48–6.08 | - |
| LMM | 3 | 0.67 | 0.05–0.95 | NEs | 1.58–NEs | - |
| PFS, progression-free survival; CI, confidence interval; PS, performance status; WT, wild type; ALM, acral lentiginous melanoma; LMM, lentigo maligna melanoma; Mucosal, mucosal melanoma; NM, nodular melanoma; SSM, superficial spreading melanoma; irAEs, immune-related adverse events; NLR, neutrophil-to-lymphocyte ratio; LDH, lactate dehydrogenase; CRP, C-reactive protein; NEs, not estimable  ^a^Median NLR was 2.79. | | | | | | |

**Supplementary Table 3.** Response rate by baseline characteristics assessed by investigators

|  | n | CR | PR | SD | PD | NE | ORR | 95% CI | p-value |
| --- | --- | --- | --- | --- | --- | --- | --- | --- | --- |
| All patients | 124 | 3  (2.4) | 19  (15.3) | 29  (23.4) | 58  (46.8) | 15  (12.1) | 22  (17.7) | 11.5–25.6 |  |
| Sex |  |  |  |  |  |  |  |  |  |
| Male | 72 | 2  (2.8) | 13  (18.1) | 15  (20.8) | 32  (44.4) | 10  (13.9) | 15  (20.8) | 12.2–32.0 | 0.346 |
| Female | 52 | 1  (1.9) | 6  (11.5) | 14  (26.9) | 26  (50.0) | 5  (9.6) | 7  (13.5) | 5.6–25.8 |  |
| Age, years |  |  |  |  |  |  |  |  |  |
| ≥65 | 79 | 3  (3.8) | 11  (13.9) | 21  (26.6) | 35  (44.3) | 9  (11.4) | 14  (17.7) | 10.0–27.9 | 1.0 |
| <65 | 45 | 0  (0.0) | 8  (17.8) | 8  (17.8) | 23  (51.1) | 6  (13.3) | 8  (17.8) | 8.0–32.1 |  |
| PS |  |  |  |  |  |  |  |  |  |
| 0 | 79 | 3  (3.8) | 14  (17.7) | 24  (30.4) | 36  (45.6) | 2  (2.5) | 17  (21.5) | 13.1–32.2 | 0.221^a^ |
| 1 | 35 | 0  (0.0) | 5  (14.3) | 5  (14.3) | 19  (54.3) | 6  (17.1) | 5  (14.3) | 4.8–30.3 |  |
| ≥2 | 10 | 0  (0.0) | 0  (0.0) | 0  (0.0) | 3  (30.0) | 7  (70.0) | 0 | - |  |
| *BRAF* at baseline |  |  |  |  |  |  |  |  |  |
| WT | 93 | 2  (2.2) | 15  (16.1) | 25  (26.9) | 42  (45.2) | 9  (9.7) | 17  (18.3) | 11.0–27.6 | 0.189^b^ |
| Mutant | 20 | 1  (5.0) | 0  (0.0) | 1  (5.0) | 13  (65.0) | 5  (25.0) | 1  (5.0) | 0.1–24.9 |  |
| Unknown | 10 | 0  (0.0) | 4  (40.0) | 3  (30.0) | 2  (20.0) | 1  (10.0) | 4  (40.0) | 12.2–73.8 |  |
| Histopathological classification | | | |  |  |  |  |  |  |
| Mucosal | 42 | 0  (0.0) | 8  (19.0) | 8  (19.0) | 21  (50.0) | 5  (11.9) | 8  (19.0) | 8.6–34.1 | 0.807 |
| NM | 19 | 1  (5.3) | 3  (15.8) | 1  (5.3) | 11  (57.9) | 3  (15.8) | 4  (21.1) | 6.1–45.6 | 0.745 |
| SSM | 16 | 1  (6.3) | 1  (6.3) | 3  (18.8) | 8  (50.0) | 3  (18.8) | 2  (12.5) | 1.6–38.3 | 0.735 |
| ALM | 25 | 1  (4.0) | 3  (12.0) | 10  (40.0) | 9  (36.0) | 2  (8.0) | 4  (16.0) | 4.5–36.1 | 1.0 |
| Unclassified | 10 | 0  (0.0) | 1  (10.0) | 2  (20.0) | 4  (40.0) | 3  (30.0) | 1  (10.0) | 0.3–44.5 | 0.690 |
| Others | 55 | 0  (0.0) | 11  (20.0) | 13  (23.6) | 26  (47.3) | 5  (9.1) | 11  (20.0) | 10.4–33.0 | 0.639 |
| Brain metastasis |  |  |  |  |  |  |  |  |  |
| Absent | 110 | 3  (2.7) | 15  (13.6) | 28  (25.5) | 54  (49.1) | 10  (9.1) | 18  (16.4) | 10.0–24.6 | 0.271 |
| Present | 14 | 0  (0.0) | 4  (28.6) | 1  (7.1) | 4  (28.6) | 5  (35.7) | 4  (28.6) | 8.4–58.1 |  |
| LDH, IU/L |  |  |  |  |  |  |  |  |  |
| <400 | 92 | 3  (3.3) | 15  (16.3) | 23  (25.0) | 46  (50.0) | 5  (5.4) | 18  (19.6) | 12.0–29.1 | 0.588 |
| ≥400 | 30 | 0  (0.0) | 4  (13.3) | 4  (13.3) | 12  (40.0) | 10  (33.3) | 4  (13.3) | 3.8–30.7 |  |
| CRP, mg/dL |  |  |  |  |  |  |  |  |  |
| <1.0 | 79 | 2  (2.5) | 12  (15.2) | 23  (29.1) | 38  (48.1) | 4  (5.1) | 14  (17.7) | 10.0–27.9 | 1.0 |
| ≥1.0 | 39 | 0  (0.0) | 7  (17.9) | 4  (10.3) | 18  (46.2) | 10  (25.6) | 7  (17.9) | 7.5–33.5 |  |
| NLR |  |  |  |  |  |  |  |  |  |
| <median^c^ | 61 | 2  (3.3) | 8  (13.1) | 20  (32.8) | 29  (47.5) | 2  (3.3) | 10  (16.4) | 8.2–28.1 | 1.0 |
| ≥median^c^ | 62 | 0  (0.0) | 11  (17.7) | 9  (14.5) | 29  (46.8) | 13  (21.0) | 11  (17.7) | 9.2–29.5 |  |
| Data are presented as n (%) unless otherwise indicated.  CR, complete response; PR, partial response; SD, stable disease; PD, progressive disease; NE, not evaluable; ORR, objective response rate; CI, confidence interval; PS, performance status; WT, wild type; ALM, acral lentiginous melanoma; Mucosal, mucosal melanoma; NLR, neutrophil-to-lymphocyte ratio; NM, nodular melanoma; SSM, superficial spreading melanoma; LDH, lactate dehydrogenase; CRP, C-reactive protein  ^a^PS of 0 vs. ≥1; ^b^WT *BRAF* vs. mutated *BRAF* and unknown; ^c^Median NLR was 2.79. | | | | | | | | | |

**Supplementary Table 4.** Response rates by treatment line and immune-related adverse events assessed by investigators

|  | n | CR | PR | SD | PD | NE | ORR | 95% CI | p-value^a^ |
| --- | --- | --- | --- | --- | --- | --- | --- | --- | --- |
| All patients | 124 | 3  (2.4) | 19  (15.3) | 29  (23.4) | 58  (46.8) | 15  (12.1) | 22  (17.7) | 11.5–25.6 |  |
| Treatment line |  |  |  |  |  |  |  |  |  |
| First-line | 77 | 2  (2.6) | 16  (20.8) | 18  (23.4) | 34  (44.2) | 7  (9.1) | 18  (23.4) | 14.5–34.4 | 0.0512 |
| Second-line | 47 | 1  (2.1) | 3  (6.4) | 11  (23.4) | 24  (51.1) | 8  (17.0) | 4  (8.5) | 2.4–20.4 |  |
| irAEs |  |  |  |  |  |  |  |  |  |
| Not reported | 67 | 0  (0.0) | 3  (4.5) | 14  (20.9) | 35  (52.2) | 15  (22.4) | 3  (4.5) | 0.9–12.5 | 0.00003 |
| Reported | 57 | 3  (5.3) | 16  (28.1) | 15  (26.3) | 23  (40.4) | 0  (0.0) | 19  (33.3) | 21.4–47.1 |  |
| irAEs specified (including overlap) | | | |  |  |  |  |  |  |
| Skin disorders | 22 | 1  (4.5) | 10  (45.5) | 3  (13.6) | 8  (36.4) | 0  (0.0) | 11  (50.0) | 28.2–71.8 | 0.0001^b^  0.0460^c^ |
| Vitiligo^d^ | 14 | 1  (7.1) | 7  (50.0) | 4  (28.6) | 2  (14.3) | 0  (0.0) | 8  (57.1) | 28.9–82.3 | 0.0004^b^  0.0487 ^c^ |
| Thyroid dysfunctions | 21 | 3  (14.3) | 5  (23.8) | 7  (33.3) | 6  (28.6) | 0  (0.0) | 8  (38.1) | 18.1-61.6 | 0.0127^b^  0.575^c^ |
| Hypothyroidism | 13 | 1  (7.7) | 5  (38.5) | 5  (38.5) | 2  (15.4) | 0  (0.0) | 6  (46.2) | 19.2–74.9 | 0.0119^b^  0.323^c^ |
| Hyperthyroidism | 8 | 2  (25.0) | 0  (0.0) | 2  (25.0) | 4  (50.0) | 0  (0.0) | 2  (25.0) | 3.2–65.1 | 0.631^b^  0.706^c^ |
| Impaired liver function | 5 | 0  (0.0) | 0  (0.0) | 1  (20.0) | 4  (80.0) | 0  (0.0) | 0 | - | 0.585^b^  0.158^c^ |
| Interstitial pneumonia | 3 | 0  (0.0) | 1  (33.3) | 1  (33.3) | 1  (33.3) | 0  (0.0) | 1  (33.3) | 0.8–90.6 | 0.446^b^  1.0^c^ |
| Colitis | 3 | 0  (0.0) | 0  (0.0) | 0  (0.0) | 3  (100.0) | 0  (0.0) | 0 | - | 1.0 ^b^  0.544^c^ |
| Hypophysitis/ hypopituitarism | 3 | 0  (0.0) | 1  (33.3) | 1  (33.3) | 1  (33.3) | 0  (0.0) | 1  (33.3) | 0.8–90.6 | 0.446^b^  1.0^c^ |
| Adrenal insufficiency | 2 | 0  (0.0) | 1  (50.0) | 1  (50.0) | 0  (0.0) | 0  (0.0) | 1  (50.0) | 1.3–98.7 | 0.325^b^  1.0^c^ |
| Type 1 diabetes mellitus | 1 | 0  (0.0) | 0  (0.0) | 0  (0.0) | 1  (100.0) | 0  (0.0) | 0 | - | 1.0 ^b^  1.0^c^ |
| Other irAEs | 15 | 1  (6.7) | 2  (13.3) | 5  (33.3) | 7  (46.7) | 0  (0.0) | 3  (20.0) | 4.3–48.1 | 0.729^b^  0.339^c^ |
| Data are presented as n (%) unless otherwise indicated.  CR, complete response; PR, partial response; SD, stable disease; PD, progressive disease; NE, not evaluable; ORR, objective response rate; CI, confidence interval; irAEs, immune-related adverse events  ^a^by two-sided Fisher’s exact test; ^b^difference in the ORR between subgroups with and without irAEs of interest in the full analysis set (n = 124); ^c^difference in the ORR between subgroups with and without irAEs of interest within patients in whom any irAEs were observed (n = 57); ^d^Depigmentation of skin | | | | | | | | | |


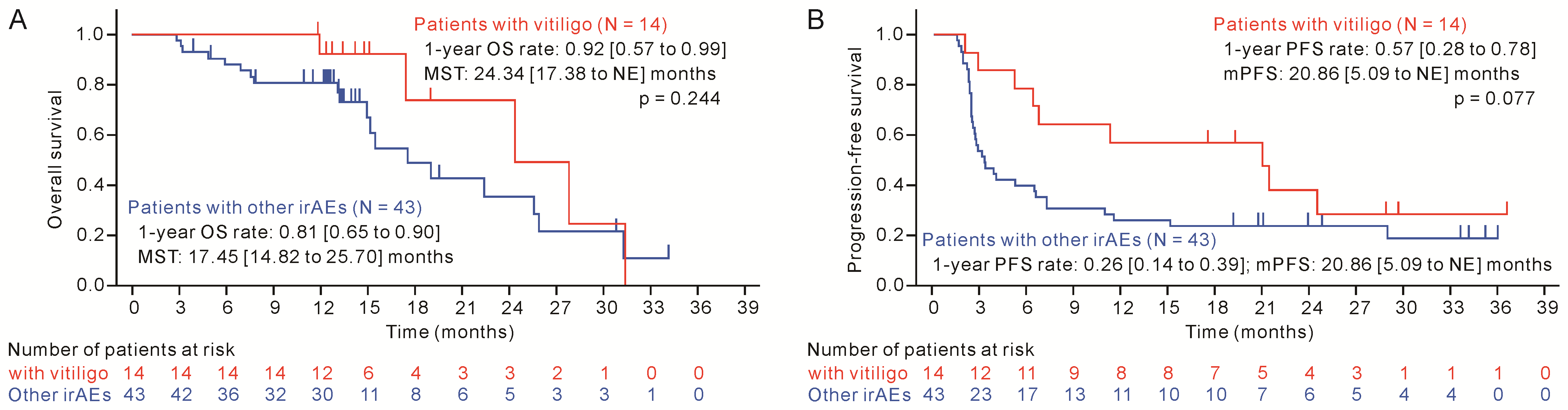


**Supplementary Figure 1.** Survival analysis comparing patients with and without vitiligo among patients who developed immune-related adverse events. (A) OS. (B) PFS. One-year OS rate, MST, 1-year PFS, mPFS, and their 95% CIs [lower to upper bound] were estimated by Kaplan–Meier analysis. All p-values were computed by a two-sided log-rank test. OS, overall survival; PFS, progression-free survival; MST, median survival time; mPFS, median progression-free survival; CI, confidence interval; NE, not estimable.


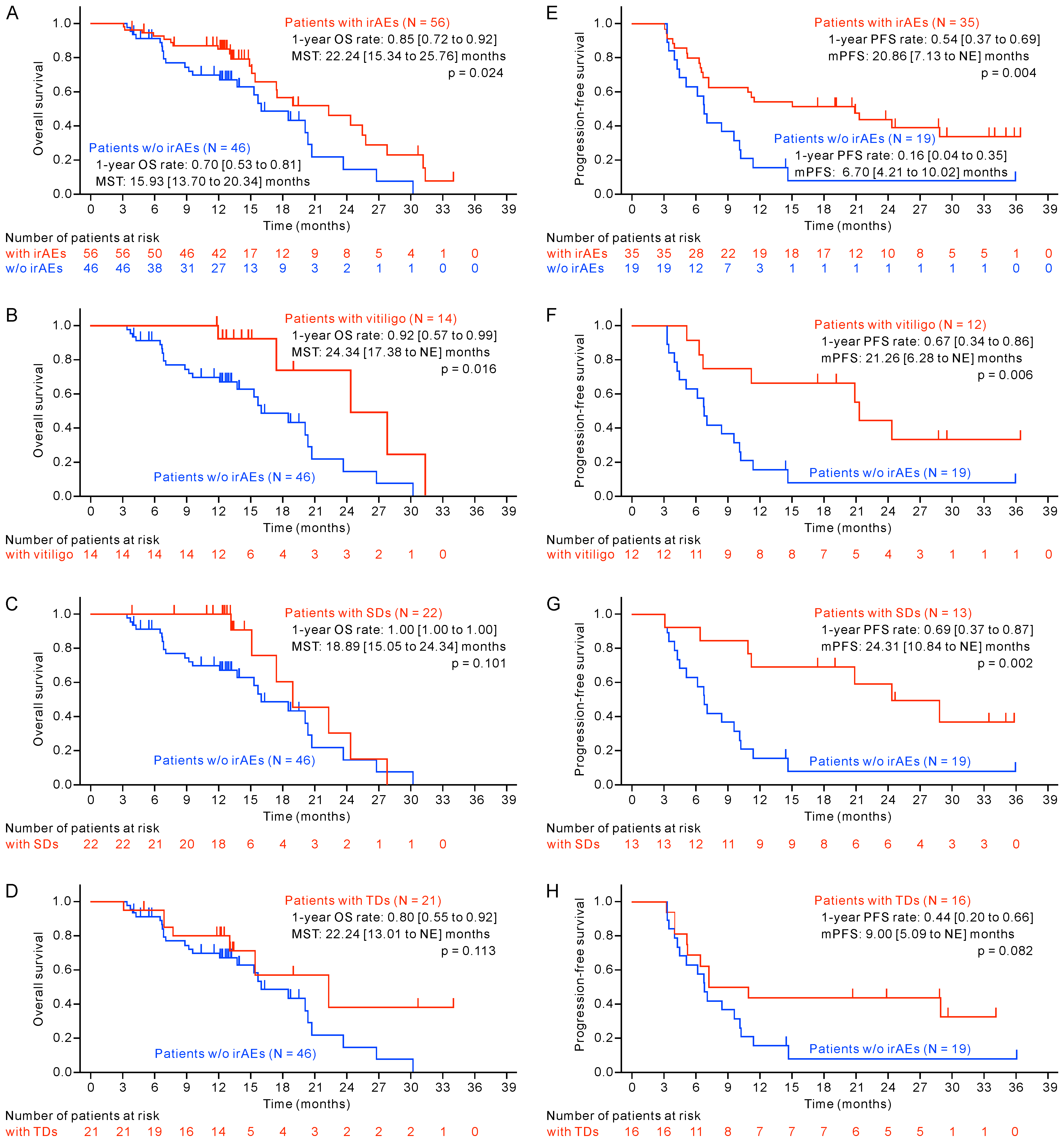


**Supplementary Figure 2.** Landmark survival analysis between subgroups with and without irAEs. OS and PFS were compared by 3-month landmark analysis between patient subgroups without irAEs and with any irAEs (A and E), without and with vitiligo (B and F), without and with SDs (C and G), and without and with TD (D and H). The 1-year OS rate, MST, 1-year PFS, mPFS, and their 95% CIs [lower to upper bound] were estimated by Kaplan–Meier analysis. All p-values were computed by a two-sided log-rank test. irAEs, immune-related adverse events; OS, overall survival; PFS, progression-free survival; SDs, skin disorders; TD, thyroid dysfunctions; MST, median survival time; mPFS, median progression-free survival; CI, confidence interval; NE, not estimable.
